# Colloidal silver against macrophage infections and biofilms of atypical mycobacteria

**DOI:** 10.1007/s10534-023-00494-w

**Published:** 2023-02-02

**Authors:** Sholeh Feizi, Clare M. Cooksley, Mahnaz Ramezanpour, Roshan Nepal, Alkis J. Psaltis, Peter-John Wormald, Sarah Vreugde

**Affiliations:** 1 Department of Surgery-Otolaryngology Head and Neck Surgery, Basil Hetzel Institute for Translational Health Research, Central Adelaide Local Health Network, Woodville South, Australia; 2 The University of Adelaide, Adelaide, Australia

**Keywords:** Green synthesis, Colloidal silver, Atypical mycobacteria, Skin infection, Macrophages

## Abstract

Skin and soft tissue infection (SSTI) caused by atypical mycobacteria such as *Mycobacterium abscessus* and *Mycobacterium avium* intracellulare complex (MAIC) have increased in recent years. Current therapeutic options are limited, and hence new and better therapies are urgently required. Colloidal Silver (CS) has been identified for its widespread antibacterial properties and silver-impregnated dressings have been used for SSTIs caused by various pathogens. The efficacy of Green Synthesized Colloidal Silver (GSCS) was investigated for bacterial growth inhibition (BGI) using a microdilution method and minimum biofilm eradication concentration (MBEC) using resazurin assay and confocal scanning laser microscopy (CSLM) of *M. abscessus* (n = 5) and MAIC (n = 5). The antibacterial effect of GSCS against *M. abscessus* infected macrophages was also evaluated. The in vitro cytotoxicity of GSCS on a human keratinocyte cell line (HaCaT) and neonatal foreskin fibroblasts was analyzed by the crystal violet proliferation assay. Average BGI and MBEC of GSCS varied between 0.7 and 22 ppm for *M. abscessus* and MAIC. The concentration of 3 ppm reduced *M. abscessus*-infection in macrophages significantly. GSCS was not cytotoxic to HaCaT and neonatal foreskin fibroblast cells at concentrations < 3 ppm up to 2 h exposure time. GSCS therefore, has the potential for topical application against atypical mycobacterial SSTI.

## Introduction

Nontuberculous mycobacteria (NTM) along with *Mycobacterium tuberculosis* complex and *Mycobacterium leprae* belong to the family of Actinobacteria and the genus of Mycobacteria (Misch et al. 2018). NTM are also called environmental mycobacteria, or atypical mycobacteria and are abundant in nature in plants, animals, soil and water (Gonzalez-Santiago and Drage 2015).

NTM can cause difficult to treat lung infections in susceptible individuals and skin and soft tissue infections (SSTIs) where they usually present as abscesses, sporotrichoid nodules or ulcers (Misch et al. 2018). These SSTIs typically occur after trauma, surgery and cosmetic procedures and their prevalence has been increasing in recent years (Misch et al. 2018). NTM can also form biofilms and internalize within macrophages and have intrinsic or inducible resistance to most antibiotics and immune system surveillance systems (Gonzalez-Santiago and Drage 2015; Ananta et al. 2018; Kim et al. 2019).

Amongst the > 170 NTM species reported, *M. abscessus* is regarded as the most pathogenic and clinically challenging member (Lee et al. 2015). It can cause SSTIs after direct contact of damaged skin to contaminated water or soil. These infections can also occur after various procedures often involving the insertion of contaminated surgical instruments or needles such as Mohs surgery, liposuction, soft tissue augmentation, mesotherapy, injections and acupuncture (Wallace et al. Jr 1983; Murillo et al. 2000; Fisher and Gloster Jr 2005; Ryu et al. 2005; Uslan et al. 2006; Garcia-Navarro et al. 2008; Wongkitisophon et al. 2011; Lee et al. 2015). Treatment can be challenging and often involves a combination of surgical excision and 3–6 months’ administration of antibiotics such as clarithromycin in combination with amikacin, cefoxitin, tigecycline, or imipenem (Griffith et al. 2007; Esteban and Ortiz-Pérez 2009; Wang and Pancholi 2014).

*Mycobacterium avium* intracellulare complex (MAIC) is the most ubiquitous species of NTM and is the main driver for the rise in mycobacteriosis worldwide and can also cause skin infections (Lee et al. 2015; Busatto et al. 2019). MAIC can be internalized within macrophages and evade the immune system along with their ability to form biofilm making MAIC infections difficult to treat (Schorey and Sweet 2008; Yamazaki et al. 2006). Cutaneous infections can present as nodules, plaques, or ulcers. The standard treatment of MAIC skin infection is systemic antibiotics with or without surgical excision (Dyer et al. 2016).

MAIC is an acid-fast slow-growing mycobacterium (SGM), classified as non-chromogens in group III of the Runyon classification of NTM, while *M. abscessus* is a rapid-growing mycobacterium (RGM) (To et al. 2020). MAIC is a non-pathogenic opportunistic NTM, whereas, *M. abscessus* is a highly pathogenic NTM (To et al. 2020). The pathogenesis of MAIC involves its ability to colonize and invade intestinal or respiratory mucosa to infect phagocytic cells (To et al. 2020). However, the pathogenesis of *M. abscessus* is related to biofilm formation and transformation from a colonizing lycopeptidolipids-expressing smooth phenotype that evades innate immune responses to a virulent lycopeptidolipids-deficient rough phenotype, capable of causing extensive tissue damage (To et al. 2020).

NTM are known to have high resistance rates to antibiotics and even disinfectants due to their lipid-rich cell wall which acts like a barrier preventing drugs from entering the cell (Jarlier and Nikaido 1994). They are also equipped with efflux systems which are protein pumps in the bacterial cell wall pumping antibiotics out of the bacteria (Jarlier and Nikaido 1994; da Silva et al. 2011; Ramón-García et al. 2009). Therefore, novel treatment strategies to fight mycobacterial infections are required, one of which is colloidal silver (CS) due to its antimicrobial properties (Ansari et al. 2011).

Disrupting cell walls and membranes, CS enters the bacterial cells, inducing the production of reactive oxygen species (ROS), which disturbs signal transduction leading to the cell death (Dakal et al. 2016). CS binds to phosphorus or sulfur containing elements such as DNA and proteins disrupting their function (Wasilewska et al. 2023). CS can be produced by chemical, physical and biological methods (Wasilewska et al. 2023). The biological methods contribute significantly to the production of CS because of the use of environmentally friendly reagents as both reducing and stabilizing agents (Wasilewska et al. 2023). Instead, production of CS by chemical and physical methods involves harmful by-products and high operational costs, respectively (Mittal et al. 2013; Dhas et al. 2014). Different plants and their parts have been used for the production of CS (Feizi et al. 2018; Abbasi et al. 2017). However, producing small CS particles which are stable for a long time and retain their antimicrobial properties is a matter of concern. *Corymbia maculata* (spotted gum) is one of the 700 species of gum trees which are fast growing and native to Australia. The leaf extract of *Corymbia maculata* was previously used for the production of green synthesised CS (GSCS) in an environmentally friendly and rapid 15-minute one-step process (Feizi et al. 2021a). Our previous research has indeed shown that the GSCS is 40 nm in size and highly stable for over one year at room temperature (Feizi et al. 2021c). It has antibacterial efficacy against clinical isolates like *Pseudomonas aeruginosa* and *Staphylococcus aureus* (Feizi et al. 2021a, b).

In the present study, we aim to evaluate the antimycobacterial activity of GSCS against *M. abscessus* and MAIC clinical isolates in planktonic and biofilm form as well as mycobacteria internalized within macrophages. Moreover, GSCS safety was determined on both spontaneously immortalized human keratinocyte (HaCaT) and neonatal foreskin fibroblast cell lines.

## Materials and methods

### Green synthesized colloidal silver fabrication and preparation

Green Synthesized Colloidal silver (GSCS) was produced by *Corymbia maculata* (spotted gum) aqueous leaf extract and prepared as detailed previously (Feizi et al. 2021a). In short, the leaves were cut into pieces and boiled in sterile Milli Q water (Merck Millipore, Darmstadt, Germany). Afterwards, the aqueous extract was added to pre-warmed silver nitrate solution (1 mM AgNO_3_, 99.99% trace metals basis, Sigma-Aldrich, Steinheim, Germany) and incubated at room temperature overnight without agitation. The GSCS was centrifuged at 38,500 × *g* for 1 h, 4 °C (Beckman Coulter’s Avanti JXN-26 high-speed centrifuge) and resuspended in 18 ml Milli Q water to obtain the GSCS at the concentration of 350 ppm. Finally, the solution was sonicated for 15 min [Branson sonifier 450 [Timer: hold, Duty cycle%: 40% and Output Control: 4 (80 watts)].

### Bacterial strains and cell lines

The human research ethics committee of The Queen Elizabeth Hospital (TQEH) approved the use of clinical isolates for this study (HREC/15/TQEH/132). Clinical isolates were collected from cystic fibrosis (CF) patients after written informed consent was obtained (Table 1). Isolates were identified as *Mycobacterium abscessus* and MAIC by an independent diagnostic microbiology laboratory using matrix assisted laser desorption ionization-time of flight mass spectrometry (MALDI-TOF MS, Bruker® MBT) (SA Pathology, Adelaide, Australia). THP-1 cells (human monocytic cells) were purchased from American Type Culture Collection (ATCC, Manassas, USA). The HaCaT cell line and neonatal foreskin fibroblasts were kindly provided by Dr. Louise Smith (Mawson Lakes, University of South Australia, Adelaide, Australia), and Dr. Prue Cowled (Discipline of Surgery, The University of Adelaide, Basil Hetzel Institute, The Queen Elizabeth Hospital).

**Table 1:** Bacterial isolates for bacterial growth inhibition (BGI) and minimum biofilm eradication concentration (MBEC) assays

Bacterium	Number of isolates used in BGI and MBEC assays	Isolate ID	Isolate origin
*M. abscessus*	5	064, 065, 066, 071 and 073	CF patients
MAIC	5	084, 085, 086, 087 and 089	CF patients

*M. abscessus*  *mycobacterium abscessus*, *MAIC * *mycobacterium avium* intracellular complex

### Bacterial growth inhibition

Clinical isolates of *M. abscessus* and MAIC were streaked out on blood agar plates (Thermofisher, Australia) and Lowenstein-Jensen agar slopes (Thermofisher, Australia), respectively. The antibacterial effect of GSCS against the planktonic form of bacteria was performed by the microdilution method as described by the Clinical and Laboratory Standard Institute (Clinical and Institute 2006). Briefly, one colony of bacteria was suspended in Tryptone Soy Broth (TSB, for *M. abscessus*) or Middlebrook 7H9 (BD Life Sciences North Ryde NSW Australia) broth supplemented with 10% OADC (oleic acid–albumin–dextrose–catalase, for MAIC) to adjust the turbidity to 0.5 ± 0.1 McFarland units (approximately 1.5 × 10^8^ CFU/ml) and 1 ± 0.1 McFarland units (approximately 3 × 10^8^ CFU/ml) for *M. abscessus* and MAIC, respectively. Following a 1:100 dilution in respective broth, 100 µl was added to 96-well plates (Costar, Corning Incorporated, Corning, USA) along with 100 µl of different concentrations of GSCS, then the plates were incubated at 37 °C with 5% CO_2_ for 5 and 7 days for *M. abscessus* and MAIC, respectively. Finally, the absorbance was read at 595 nm on a microplate reader (Imark plate reader, Bio-Rad). The bacterial growth inhibition percentage (BGI%) of GSCS was measured according to Eq. 1.

1 $$BGI\%=100-(\frac{{A}_{T}}{{A}_{C}}\times 100\%)$$

Antibacterial activity of GSCS on the planktonic form of bacteria is expressed as bacterial growth inhibition percentage (BGI%), where A_C_ is the absorbance of the untreated control bacteria (100% bacterial growth) and A_T_ is the absorbance observed in the treated bacteria. Both A_C_ and A_T_ were corrected for background absorbance (broth only). The effect of GSCS against growth inhibition of planktonic bacteria was performed independently three times with three wells per treatment.

### Biofilm eradication

The effect of GSCS on biofilm eradication was determined by the microdilution method (Reller et al. 2009). Briefly, one colony of bacteria was resuspended in 0.9% NaCl to adjust the turbidity to 2.0 ± 0.1 McFarland units (approximately 6 × 10^8^ CFU/ml) and 3.0 ± 0.1 McFarland units (approximately 9 × 10^8^ CFU/ml) for *M. abscessus* and MAIC, respectively. The bacterial suspensions were diluted 1:15 in TSB and Middlebrook 7H9 broth, supplemented with egg yolk, ADC (bovine albumin fraction, dextrose and catalase), mycobactin J (Allied Monitor Fayette, MO, U.S.A) and PANTA (BD Life Sciences North Ryde NSW Australia) for *M. abscessus* and MAIC, respectively (Whittington et al. 2013; Ali et al. 2019). Then, black 96-well plates (Costar, Corning Incorporated, Corning, USA) were filled with 150 µl of diluted bacterial suspension per well and incubated at 37 °C with 5% CO_2_ for 5 and 14 days for *M. abscessus* and MAIC, respectively, on a rotating platform at 70 rpm. After washing twice with Phosphate Buffered Saline (PBS), biofilms were exposed to different concentrations of GSCS (3–75 ppm) for 1 h at 37 °C with 5% CO_2_ on a rotating platform at 70 rpm. The biofilm treated with the broth was considered the positive control. Plates were washed twice with sterile water to remove excess GSCS followed by alamarBlue assays (Life Technologies, Australia) following the manufacturer’s instructions. Briefly, the wells were filled with 200 µl of resazurin prepared at the concentration of 10% (in broth) and incubated at 37 °C with 5% CO_2_ on a rotating platform at 70 rpm in the dark. The fluorescence intensity was measured every hour until reaching maximum fluorescence on a FLUOstar OPTIMA plate reader at λ excitation = 530 nm/λ emission = 590 nm. Biofilm eradication percentage (BE%) was measured according to Eq. 2.

2 $$BE\%=100-(\frac{{F}_{T}}{{F}_{C}}\times 100\%)$$

Green synthesized colloidal silver (GSCS) biofilm eradication capability is expressed as the biofilm eradication percentage (BE%), where F_C_ is the fluorescence of the untreated control biofilm (100% biofilm viability) and F_T_ is the fluorescence observed in the treated biofilm. Both F_C_ and F_T_ were corrected for background fluorescence (broth only). Biofilm eradication studies were performed independently three times with four wells per treatment.

#### Confocal scanning laser microscopy

The biofilm of *M. abscessus* (064) was grown on a Cell Imaging slide (Eppendorf, Australia, Cat: 0030742079) as described in the previous section. Different concentrations of GSCS (44, 22 and 11 ppm) were applied for 1 h and biofilm was fixed using 5% glutaraldehyde (Sigma Aldrich) in water for 10 min at room temperature. The excess fixative was removed by washing with 0.9% NaCl. Then the biofilm was stained by using LIVE/DEAD® BacLightTM Bacterial Viability Kit (BacLight Kit, Invitrogen Molecular Probes, Mulgrave, Victoria, Australia) which is dual staining of SYTO9 and propidium iodide (PI) fluorescent dyes differentiating live and dead bacteria based on the membrane integrity (Gantenbein-Ritter et al. 2011). To stain the biofilm, 1.5 µl of each dye was diluted in 1 ml MilliQ water (Millipore, Billerica, MA, USA) and 200 µl was applied to the samples, followed by incubation at room temperature for 15 min. The excess stain was removed using 0.9% NaCl and the chamber slide was mounted with coverslips. All the steps were carried out protected from light. Imaging was performed using a confocal laser scanning microscope (LSM700; Carl Zeiss, Jena, Germany).

### Differentiated THP-1 infection and treatment

THP-1 cell line was grown in Roswell Park Memorial Institute medium (RPMI, Gibco, Invitrogen, Melbourne, Australia) supplemented with 1: 1 ratio of streptomycin, penicillin (50 U/mL, Thermo Fisher, Australia), L-glutamine (2 mM, Thermo Fisher, Australia) and Fetal Bovine Serum (10% FBS, qualified, heat inactivated, Thermo Fisher, Australia) in a fully humidified incubator with 5% CO_2_ at 37 °C (Richter et al. 2017). THP-1 cells at 0.15 × 10^6^ cells/ml (1500 µl) in RPMI supplemented with 0.9% NaCl, L-glutamine and FBS containing 200 ng/ml phorbol 12-myristate 13-acetate (PMA) were seeded in a 12-well plate and incubated for 48 h at 37 °C with 5% CO_2_. Then, cells were infected with *M. abscessus* (064) in a ratio of 1:10 (cell: bacteria) for 5 h. Following washing twice with PBS, the cells were treated with different concentrations of GSCS (3, 5.5, 11 and 22 ppm) in water and RPMI supplemented with 0.9% NaCl, L-glutamine (2 mM, Thermo Fisher, Australia) and 10% FBS for 1 h. Uninfected and infected cells treated with water and RPMI supplemented with 0.9% NaCl, L-glutamine and FBS were considered negative and positive controls, respectively. The cells were then incubated in a fully humidified with 5% CO_2_ at 37 °C with 200 ug/ml amikacin in media for 2 h to kill any extracellular bacteria. Finally, they were exposed to 1% Triton X-100 for 1 h to lyse the cells, and the contents of each well were serially diluted in 0.9% NaCl, plated onto blood agar and CFU counted after 4 days incubation in a fully humidified incubator with 5% CO_2_ at 37 °C.

### Cytotoxicity studies

HaCaT cells and neonatal foreskin fibroblasts were grown in Dulbecco’s Modified Eagle’s Medium (DMEM, high glucose, HEPES, 12,430,054, Thermo Fisher) supplemented with a 1:1 ratio of streptomycin, penicillin (50 U/mL, Thermo Fisher, Australia), L-glutamine (2 mM, Thermo Fisher, Australia) and Fetal Bovine Serum (10% FBS, qualified, heat inactivated, Thermo Fisher, Australia) in a fully humidified incubator with 5% CO_2_ at 37 °C (Colombo et al. 2017; Huang et al. 2013).

To perform the toxicity assay, 100 µl of 0.01 × 10^6^ cells/well were seeded in a 96-well plate and incubated overnight. The cells were exposed to different concentrations of GSCS (0.7–175 ppm) for different times (1 h, 2 and 24 h), followed by the determination of the cell viability using a crystal violet proliferation assay (Palethorpe et al. 2017).

Briefly, cells were fixed in 10% neutral buffered formalin for 30 min. Then they were stained with 1% crystal violet in 2% ethanol for 10 min. After washing with distilled water, the plates were incubated with 10% acetic acid on a rocking platform at room temperature for 1 h to elute the crystal violet. Finally, the absorbance was read at 595 nm on a microplate reader (Imark plate reader, Bio-Rad). Cell viability percentage (CV%) was quantified according to Eq. 3.

3 $$CV\%=\frac{{A}_{T}}{{A}_{C}}\times 100\%$$

The amount of cell biomass was expressed as the cell viability percentage (CV%), where A_C_ is the Absorbance of the untreated control cells (100% cell viability) and A_T_ is the Absorbance.

observed in the treated cells. Both A_C_ and A_T_ were corrected for background absorbance (medium only). Viability studies were carried out independently three times with four wells per treatment.

### Statistical analysis

All experiments were performed in three independent experiments with three wells per treatment and are shown as mean ± standard deviation (SD) by one-way analysis of variance (ANOVA) (GraphPad Prism version 8.00, GraphPad Software, La Jolla, U.S.). Statistical significance was estimated at the 95% confidence level and a p-value of < 0.05 was considered statistically significant.

## Results

### Antibacterial activity studies

**Fig. 1:**
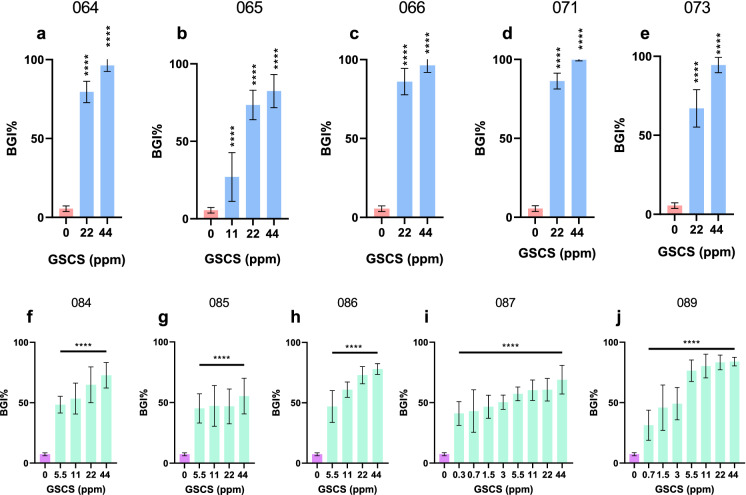
Percentage of bacterial growth inhibition (BGI%) after treatment with green synthesized colloidal silver (GSCS). BGI% of GSCS at various concentrations against *M. abscessus* clinical isolates (CI) (5 CIs- 064, 065, 066, 071 and 071, respectively shown in a-e) or MAIC clinical isolates (CI) (5 CIs- 084, 085, 086, 087 and 089, respectively shown in f–j) compared to no-treatment (negative control). Only significant results in comparison to the negative control are shown. Data represent the mean ± SD of three biological replicates, one-way analysis of variance (ANOVA). Statistical comparison to the negative control (bacteria only). ***, p < 0.001; ****, p < 0.0001;* SD* standard deviation. (Color figure online)

Green synthesized colloidal silver (GSCS) at concentrations ranging from 0.3 to 44 ppm were tested to inhibit the planktonic growth of 5 *M. abscessus* and 5 MAIC clinical isolates (Table 1). Bacterial growth inhibition of GSCS was different between bacterial species and among strains for each of the species. Overall, MAIC clinical isolates were more sensitive to GSCS treatment than *M. abscessus* with concentrations as low as 0.3 ppm GSCS having significant antimicrobial effects whilst higher concentrations of 11 ppm and above were required for significant antimicrobial effects against *M. abscessus*. Higher concentrations of 44 ppm consistently reduced both *M. abscessus* by more than 27% (range 27–100%) and MAIC by more than 31% (range 31–87%) compared to control. Significant results of bacterial growth inhibition compared to the control (untreated bacteria) are shown for *M. abscessus* and MAIC in Fig. 1.

**Fig. 2:**
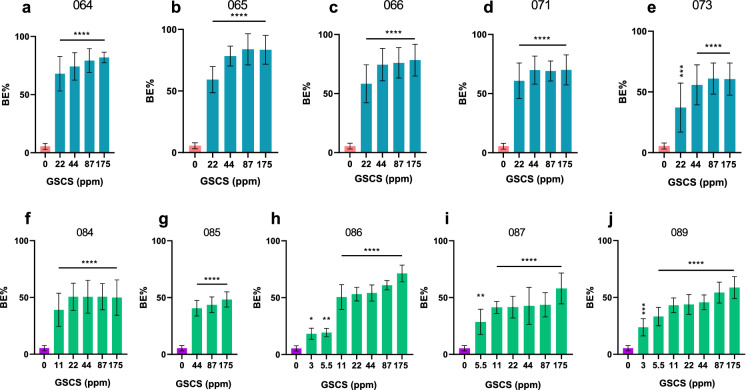
Minimum biofilm eradication concentration of green synthesized colloidal silver (GSCS). Biofilm eradication percentage (BE%) of GSCS at various concentrations against *M. abscessus* clinical isolates (CI) (5 CIs- 064, 065, 066, 071 and 071, respectively shown in a-e) or MAIC clinical isolates (CI) (5 CIs- 084, 085, 086, 087 and 089, respectively shown in f-j) compared to no-treatment (negative control). Only significant results in comparison to the negative control are shown. Data represent the mean ± SD of three biological replicates, one-way analysis of variance (ANOVA). Statistical comparison to the negative control (bacteria only). *, p < 0.05; **, p < 0.01; ***, p < 0.001; ****, p < 0.0001; * SD* standard deviation. (Color figure online)

GSCS biofilm eradication capability followed a similar trend to the planktonic assays with MAIC biofilms appearing to be more sensitive to GSCS treatment than *M. abscessus* with concentrations as low as 3 ppm GSCS having significant antibiofilm effects against MAIC whilst higher concentrations of at least 22 ppm were required for significant antibiofilm effects against *M. abscessus*. Higher concentrations of 87 ppm consistently eradicated *M. abscessus* biofilms > 33% (range 32–83%) whilst MAIC biofilm eradication reduced to > 23% (range 22–66%) at that concentration. Significant biofilm reduction compared to the positive control (untreated biofilm) is shown in Fig. 2.

#### Determination of bacterial viability in biofilm using confocal scanning laser microscopy

**Fig. 3:**
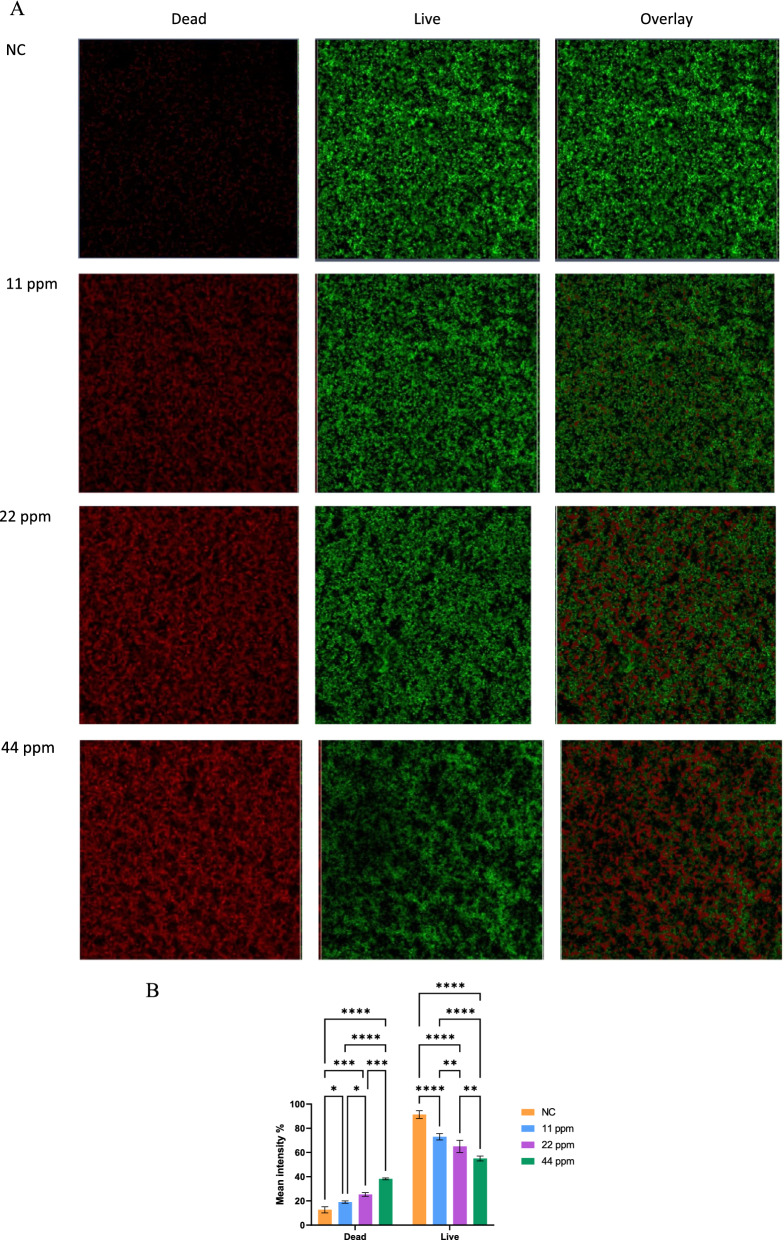
Confocal scanning laser microscopy of the treated and non-treated biofilm of *M. abscessus*. The antibiofilm activity of green synthesized colloidal silver (GSCS) on biofilm of *M. abscessus* (064) was evaluated using (A) SYTO 9 (green) and propidium iodide (red) in the absence (no- treatment, negative control (NC)) and presence of 11, 22 and 44 ppm of GSCS. All images were viewed with 20× objective power (scale bar = 50 μm). (B) Fluorescence intensity of *M. abscessus* biofilm treated with 11, 22 and 44 ppm of GSCS in comparison to no treatment (NC). The values are shown as means ± standard error of mean (SEM), * *p *< 0.05;,** p < 0.01, ***, *p <* 0.001; **** p < 0.0001, n = 3. (Color figure online)

SYTO 9 and propidium iodide are green and red fluorescent dye staining nucleic acids of the live and dead bacteria (Gantenbein-Ritter et al. 2011), respectively. SYTO 9 crosses the innate bacterial membrane, while propidium iodide enters the bacteria through disrupted membranes (Gantenbein-Ritter et al. 2011). Based on confocal scanning laser microscopy images (Fig. 3), there was a dose dependent antibiofilm effect of GSCS evidenced by an increase in the number of dead cells seen at concentrations ranging from 11 ppm to 44 ppm.

#### **Green synthesized colloidal silver efficacy against*M. abscessus*infection of macrophages**

**Fig. 4:**
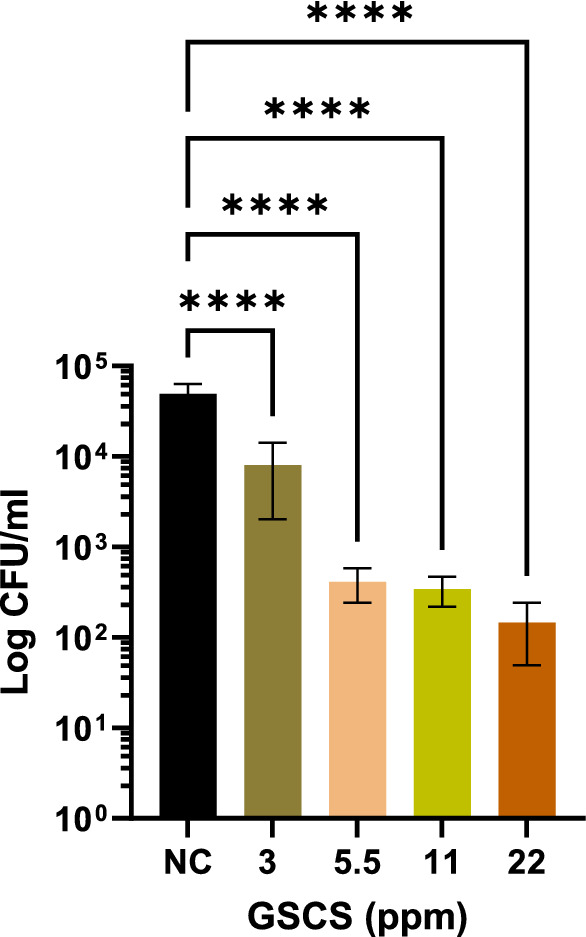
Colony forming unit (CFU) reduction after infected THP-1 treatment with green synthesized colloidal silver (GSCS) for 1 h. CFU reduction after treatment of *M. abscessus-*infected THP-1 (1 clinical isolate, 064) with GSCS. Data represent the mean ± SD of three biological replicates, one-way analysis of variance (ANOVA). Statistical comparison to the negative control (NC). **** p < 0.0001;* SD* standard deviation. (Color figure online)

A 1 h exposure to concentrations of GSCS as low as 3 ppm could kill *M. abscessus* from within differentiated THP-1 cells (p < 0.001). Significant results compared to the negative control (untreated THP-1) are shown in Fig. 4.

### Toxicity studies

**Fig. 5:**
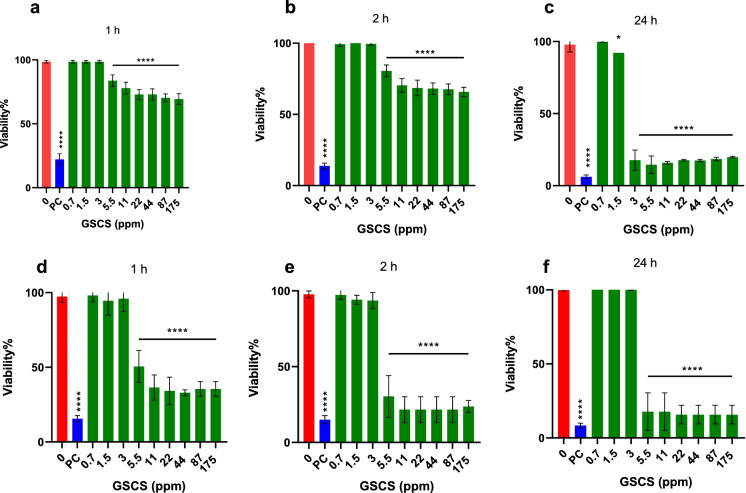
Toxicity studies in human keratinocyte (HaCaT) and neonatal foreskin fibroblasts cell lines. Crystal violet cell viability percentage of HaCaT(a, b and c) and neonatal foreskin fibroblasts (d, e and f) cell lines after 1, 2 and 24 h exposure to various concentrations of green synthesized colloidal silver (GSCS) (ppm) and 10% Triton X-100 (positive control, pc) normalized to the negative control (untreated cells). Data represent the mean ± SD of three biological replicates, one-way analysis of variance (ANOVA). Statistical comparison to the negative control. *, p < 0.05; ****, p < 0.0001;* SD* standard deviation.* PC*  positive Control. (Color figure online)

The cell viability of HaCaT and neonatal foreskin fibroblasts cell lines for different concentrations (0.7–175 ppm) of GSCS and different exposure times (1 h, 2 and 24 h) is shown in Fig. 5. The GSCS at concentrations of 0.7-3 ppm did not affect the cell viability of both cell lines in comparison to the untreated cells (p > 0.05) for up to 2 h exposure time. Although the same trend was observed for a 24 h exposure time for neonatal foreskin fibroblasts, only the lowest concentration tested (0.7 ppm GSCS) appeared to have no toxic effect on HaCaT cells after a 24 h exposure time.

## Discussion

CS and silver ions released from CS have detrimental effects on biofilm as a result of their interaction with lipids, proteins and lipopolysaccharides existing in biofilm matrix (Joshi et al. 2020). They both can penetrate through biofilm matrix and cause disruption of the biofilm and cellular components leading to biofilm eradication and bacterial death (Joshi et al. 2020). The antibiofilm effects of the GSCS used in the present study against biofilm of *P. aeruginosa*, *S. aureus* and *Haemophilus influenzae* clinical isolates was reported previously (Feizi et al. 2021a). Here, GSCS also showed efficacy against both planktonic and biofilm forms of *M. abscessus* and MAIC but was more effective against planktonic forms compared to biofilms. This is not entirely unexpected as it is well known that biofilms are in general less susceptible to antibiotics and immune defence mechanisms of the host compared to their planktonic cells. However, this is in contrast with our previous study where GSCS was more effective against *P. aeruginosa* and *S. aureus* biofilms compared to matched planktonic forms (Feizi et al. 2021a). The exact reason for this relative reduction of antibiofilm properties of GSCS when compared to activity against planktonic counterparts for MAIC and *M. abscessus* is unclear. Previous research has shown distinct properties of the biofilm ultrastructure and composition of mycobacteria (Sharma et al. 2019). This includes for example the absence of the usual exopolysaccharide matrix but rather an enrichment of shorter chain mycolic acids that may form a hydrophobic extracellular matrix (Ojha et al. 2005). Also, the growth pattern of mycobacteria biofilm is different than other pathogenic species resulting in a different biofilm ultrastructure and structure (Sharma et al. 2019). Further studies will need to be conducted to understand the mechanism of the apparent reduction of GSCS activity against mycobacteria biofilms and to map the interaction of GSCS with mycobacteria biofilm.

NTM can evade phagosomal defence mechanisms and induce inflammatory factors leading to granuloma formation at the site of infection (Johansen et al. 2020). The conversion from smooth to rough variants of NTM along with intrinsic resistance to antibiotics results in a prolonged and often complicated treatment for NTM infections (Johansen et al. 2020). It may lead to reinfection and the presence of adverse effects or failure of the treatment (Johansen et al. 2020). Metal nanoparticles overcome these challenges as they can enter the macrophages and destroy the internalized pathogens (Simões et al. 2020). More specifically, CS can enter macrophages through different mechanisms including phagocytic, non-phagocytic, flip flop mechanisms or direct penetration via ion channels (Akter et al. 2018). Once inside the macrophages, CS can kill the mycobacteria through stimulation of ROS production and activation of macrophages by the release of proinflammatory cytokines in a Nitric Oxide (NO) independent way (Mohanty et al. 2013). The efficacy of plant mediated synthesized CS on the eradication of mycobacteria internalized in macrophages has been previously reported (Singh et al. 2016). In line with those studies, here, we have shown that GSCS at different concentrations had significant antimycobacterial effects against mycobacteria present within THP-1 cells.

The toxicological burden of CS depends on various properties such as size, chemical composition, structural properties, exposure time and cell type (Medina et al. 2007; Grosse et al. 2013) (Galandáková et al. 2016). The safety of GSCS was shown against Nuli-1 epithelial cell line and primary human nasal epithelial cells, previously (Feizi et al. 2021a, c). However, in this study the cytotoxicity of GSCS was assessed against neonatal foreskin fibroblasts and HaCaT cells as those would be considered to better represent the cell types exposed to GSCS in silver dressings at the site of skin infection (Johnston et al. 2010). It has been reported that CS affects keratinocytes in both a toxic and beneficial manner, causing inhibition of keratinocyte proliferation (Poon and Burd 2004; Paddle-Ledinek et al. 2006) whilst, activating wound healing is also one of their properties (Wright et al. 2002). Moreover, Galandáková et al., have found that the toxicity of CS varied according to the cell type and the size of CS (Galandáková et al. 2016). Our study indicated that the toxicity of GSCS depended on their concentration and exposure time. Regardless, a 24-hour continuous exposure of these cells at concentrations that could effectively reduce the growth of at least some of the NTM planktonic cells and biofilms appeared to be non-toxic. Further in vivo studies are required to evaluate the potential of GSCS to be used as a therapeutic agent against NTM SSTIs.

## Conclusion

The efficacy of *Corymbia maculata* (spotted gum) mediated synthesized colloidal silver was observed against both planktonic and biofilm forms of *M. abscessus* and MAIC and macrophages infected with *M. abscessus*. GSCS did not induce significant cytotoxicity on keratinocytes (HaCaT cells) and neonatal foreskin fibroblasts at concentrations < 3 ppm within 2 h exposure time. GSCS in concentrations of 0.7 ppm and 22 ppm kills the *M. abscessus* and MAIC in planktonic and biofilm forms. The concentration of 3 ppm reduces *M. abscessus* from within infected macrophages. Whilst further in vivo studies are required, these findings support the potential for GSCS to be used as a topical application against NTM SSTIs.
